# Myelin Oligodendrocyte Glycoprotein: Deciphering a Target in Inflammatory Demyelinating Diseases

**DOI:** 10.3389/fimmu.2017.00529

**Published:** 2017-05-08

**Authors:** Patrick Peschl, Monika Bradl, Romana Höftberger, Thomas Berger, Markus Reindl

**Affiliations:** ^1^Clinical Department of Neurology, Medical University of Innsbruck, Innsbruck, Austria; ^2^Department of Neuroimmunology, Center for Brain Research, Medical University of Vienna, Vienna, Austria; ^3^Institute of Neurology, Medical University of Vienna, Vienna, Austria

**Keywords:** myelin oligodendrocyte glycoprotein, demyelination, autoantibodies, inflammation, MOG

## Abstract

Myelin oligodendrocyte glycoprotein (MOG), a member of the immunoglobulin (Ig) superfamily, is a myelin protein solely expressed at the outermost surface of myelin sheaths and oligodendrocyte membranes. This makes MOG a potential target of cellular and humoral immune responses in inflammatory demyelinating diseases. Due to its late postnatal developmental expression, MOG is an important marker for oligodendrocyte maturation. Discovered about 30 years ago, it is one of the best-studied autoantigens for experimental autoimmune models for multiple sclerosis (MS). Human studies, however, have yielded controversial results on the role of MOG, especially MOG antibodies (Abs), as a biomarker in MS. But with improved detection methods using different expression systems to detect Abs in patients’ samples, this is meanwhile no longer the case. Using cell-based assays with recombinant full-length, conformationally intact MOG, several recent studies have revealed that MOG Abs can be found in a subset of predominantly pediatric patients with acute disseminated encephalomyelitis (ADEM), aquaporin-4 (AQP4) seronegative neuromyelitis optica spectrum disorders (NMOSD), monophasic or recurrent isolated optic neuritis (ON), or transverse myelitis, in atypical MS and in *N*-methyl-d-aspartate receptor-encephalitis with overlapping demyelinating syndromes. Whereas MOG Abs are only transiently observed in monophasic diseases such as ADEM and their decline is associated with a favorable outcome, they are persistent in multiphasic ADEM, NMOSD, recurrent ON, or myelitis. Due to distinct clinical features within these diseases it is controversially disputed to classify MOG Ab-positive cases as a new disease entity. Neuropathologically, the presence of MOG Abs is characterized by MS-typical demyelination and oligodendrocyte pathology associated with Abs and complement. However, it remains unclear whether MOG Abs are a mere inflammatory bystander effect or truly pathogenetic. This article provides deeper insight into recent developments, the clinical relevance of MOG Abs and their role in the immunpathogenesis of inflammatory demyelinating disorders.

## Molecular Structure and Function of Myelin Oligodendrocyte Glycoprotein (MOG)

Myelin oligodendrocyte glycoprotein is a minor myelin component, with a length of 245 amino acids (AA) and a molecular weight of 26–28 kDa. It is only present in mammals and has a highly conserved nucleotide and AA structure within different species (1). The human MOG gene is located at chromosome 6 within the human leukocyte antigen (HLA) gene locus, whereas the mouse MOG gene is located on chromosome 17 within the major histocompatibility complex (MHC) gene locus (2). MOG is exclusively expressed in the central nervous system (CNS) on the surface of myelin sheaths and oligodendrocyte processes (1–3). MOG expression starts at the onset of myelination and is therefore a potential differentiation marker for oligodendrocyte maturation (4). The function of MOG is not yet fully understood, but its molecular structure and its extracellular immunoglobulin (Ig) domain indicate a possible function as a cell surface receptor or cell adhesion molecule (5). MOG belongs to the Ig superfamily, with a single extracellular immunoglobuline variable (IgV) domain, one transmembrane domain, one cytoplasmic loop, a membrane-associated region, and a cytoplasmic tail (6). Fifteen different alternatively spliced isoforms have been detected in humans. Full-length variants alpha 1 and beta 1 are found in fetal stages, whereas alternative variants are expressed in later postnatal stages (1, 6). It has been shown, that these isoforms are localized on the cell surface, in the endoplasmic reticulum, in the endocytic system, or can be found in a secreted form. The secreted form could have important effects triggering autoimmunity if released into the cerebrospinal fluid (CSF) and then drained into the periphery. The cytoplasmic tail of MOG determines the intracellular localization of the various splice forms and could play a role in intracellular signaling (6). The cross-linking of antibodies (Abs) reactive with the extracellular domain of MOG resulted in the activation of intracellular signaling cascades resulting in survival signals, changes of cytoskeletal stability, and cellular stress responses (7). MOG is highly homologous to butyrophilins which are expressed in mammary glands (8) and might cause autoimmunity by molecular mimicry (9). Furthermore, a sequence homology of MOG AA 35–55 (MOG_35–55_) to medium-sized neurofilament leads to the activation of MOG_35–55_ specific T cells (10).

Myelin oligodendrocyte glycoprotein has been implicated to be the cellular receptor for Rubella virus (11), as a ligand for DC-SIGN on antigen-expressing cells (12), and as a receptor for nerve growth factor (13). The interaction of DC-SIGN and MOG along with its correct glycosylation might keep myeloid antigen-presenting cells (APC) in an immature and tolerogenic state and thereby prevent autoimmunity (12). However, the inactivation of mouse MOG by gene targeting resulted in no clinical or histological abnormalities (14, 15).

Whereas the biological function of MOG is still not clear, its topology at the surface of myelin and oligodendrocytes and its special characteristics predict MOG to be a very important target of autoantibodies and cell-mediated immune responses in inflammatory demyelinating diseases. Initially, MOG was discovered as a dominant target of autoantibodies (they so called it M2 antigen) after immunization of guinea pigs with CNS tissue (16, 17). Numerous studies have then established an important role of MOG as autoantigen for T and B cell responses in experimental models and inflammatory demyelinating diseases.

## Autoimmune Responses Against MOG in Animal Models

The first indications that humoral factors also contribute to demyelination have been described in 1947 by Kabat et al. who observed a demyelinating effect after immunization of rhesus monkeys with heterologous rabbit or homologous brain tissue (18). In 1968, it was noted that sera from guinea pigs sensitized with whole CNS preparations have a demyelinating effect *in vitro* (19). The first indication that MOG Abs might be pathogenic followed about 10 years later when it was observed that guinea pigs immunized with the M2 protein developed Abs with demyelinating activity *in vitro* (20, 21). Then, it was shown that the monoclonal MOG-specific Ab 8–18C5 induces demyelination in Lewis (LEW) rats with experimental autoimmune encephalomyelitis (EAE) (4, 22), that guinea pigs immunized with M2 show demyelinated lesions in their CNS, and that the M2 protein is identical to MOG (16). It soon became clear that MOG Abs may be pathogenic in a large number of additional species (Table 1) (23). Further characterizations of MOG revealed that this protein is found in the oligodendrocyte membrane with a large N-terminal extracellular IgG V-like domain (8) and that N-terminal domain (AA 1–125) is responsible for the formation of demyelinating Abs (23, 24). Studies in marmoset monkeys and mice clarified that pathogenic Abs recognize conformational epitopes on the extracellularly exposed MOG domain (25–27) and that strain specific differences in mounting such anti-conformational Ab responses correlate with exacerbation of diseases (28, 29). Epitopes for encephalitogenic T cells for many different strains of mice and for LEW rats are found on the extracellular domain of MOG (30–33), but also in its transmembrane region (34, 35) (Table 2).

**Table 1:** **The effects of myelin oligodendrocyte glycoprotein (MOG)-specific antibodies (Abs)**.

Reference	Year	Findings
Trotter et al. (36)	1986	Myelin-specific Abs trigger macrophage-mediated demyelination
Linington and Lassmann (17)	1987	Ab-mediated demyelination in a chronic relapsing experimental autoimmune encephalomyelitis (EAE) in guinea pigs
Schluesener et al. (37)	1887	Monoclonal MOG Abs induced fatal relapses in a model of chronic relapsing-remitting EAE in SJL mice and enhanced acute EAE in Lewis (LEW) rats with increased inflammation and demyelination
Lassmann et al. (22)	1988	Demyelination occurs in a synergistic way between cellular (T cells) and humoral immune mechanisms
Linington et al. (4)	1988	MOG Abs augment demyelination in a myelin basic protein (MBP) T cell-mediated EAE model in LEW rats
Kerlero de Rosbo et al. (38)	1990	Monoclonal MOG Abs together with complement lead to demyelination and MBP loss in brain cells
Scolding and Compston (39)	1991	Abs mediate macrophage-dependent phagocytosis of oligodendrocytes *in vitro*
Vass et al. (40)	1992	MOG Ab-mediated demyelination is intensified by interferon-gamma
Linington et al. (41)	1992	Abs prevent tolarization effect of repeatedly induced MBP-T cell-mediated EAE and enhances demyelination
Piddlesden et al. (42)	1993	Ab-mediated demyelination is dependent on complement recruiting ability and independent on its epitope recognition
Genain et al. (43)	1995	MOG Abs facilitate demyelination in MOG-induced EAE in common marmosets
Johns et al. (44)	1995	MOG Abs lead to degradation of MBP and increased myelin protease activity
Ichikawa et al. (45)	1996	MOG_35–55_ encephalitogenic in LEW rats and a potential target for Ab-mediated demyelination
Menon et al. (46)	1997	Ab induced MBP loss and myelin destabilization by neutral proteases in human myelin
Van der Goes et al. (47)	1999	Abs to MOG play a crucial role for the phagocytosis of myelin by macrophages *in vitro*
Von Budingen et al. (25)	2002	Ab pathogenicity in marmosets is dependent on their ability to bind on conformational epitopes
Marta et al. (48)	2003	Ab cross-linking on oligodendrocyte cultures leads to the formation of lipid rafts and to a reconstitution of MOG
Bourquin et al. (28)	2003	Generation of pathogenic Abs to conformational MOG in H-2b mice is dependent on genes encoded within the major histocompatibility complex
Von Budingen et al. (49)	2004	EAE phenotype in marmosets correlates with the availability of conformational MOG Abs resulting in typical multiple sclerosis-like disease pattern. In addition Abs to MOG peptides lead to focal disease pattern in brain stem and spinal cord. MBP T cell-mediated EAE animals showed no demyelination when injected with MOG peptides. By contrast, conformational MOG Abs were more pathologic as controls
Marta et al. (26)	2005	Human but not rat MOG-induced B cell-dependent EAE in MOG primed C57BL/6 mice and Abs of hMOG immunized mice only lead to EAE formation in B cell-deficient mice. Pathogenic Abs react to conformational intact and glycosylated antigen only
Zhou et al. (50)	2006	Patient-derived MOG Abs enhance demyelination in rat EAE models
Urich et al. (51)	2006	Ab-mediated demyelination is FcR independent but completely relies on complement activation
Jagessar et al. (52)	2008	Increased Ab-dependent demyelination in marmosets immunized with murine myelin compared to myelin lacking MOG
Harrer et al. (53)	2009	Complement induced demyelination in a murine *ex vivo* model
Ohtani et al. (54)	2011	Ab titer against conformational MOG are directly associated with EAE activity and demyelination in EAE rats
Mader et al. (55)	2011	Human MOG Abs lead to complement activated cytotoxicity in HEK293A cells
de Graaf et al. (27)	2012	Correct refolding of MOG increases its pathogenicity by generating conformation-dependent MOG Abs
Dale et al. (56)	2014	Oligodendrocytes incubated with purified human MOG IgG lead to organizational disturbances of the thin filaments and microtubule cytoskeleton
Saadoun et al. (57)	2014	Patient-derived MOG IgG lead to complement-independent myelin changes and altered expression of axonal proteins, but did not trigger inflammation or cellular death
Flach et al. (58)	2016	MOG Abs boost EAE by activation of effector T cells
Kinzel et al. (59)	2016	MOG Abs are able to trigger spontaneous EAE in mice harboring endogenous MOG-specific T cells in the absence of B cells

**Table 2:** **T cell responses against myelin oligodendrocyte glycoprotein (MOG) in experimental autoimmune encephalomyelitis (EAE) animal models**.

Reference	Year	Finding
Linington et al. (33)	1993	MOG peptide (MOG_44–53_) specific T cells induce atypical EAE in Lewis (LEW) rats
Amor et al. (30)	1994	Epitope MOG_1–22_, MOG_43–57_, and MOG_134–148_ induce clinically and pathological relevant EAE, however, mild effects in AB/H mice. Epitope MOG_92–106_ is highly encephalitogenic in SJL mice
Adelmann et al. (24)	1995	N-terminal domain (MOG_1–125_) leads to demyelination in LEW rats, T cells reactive to epitope MOG_1–20_ and MOG_35–55_ are only weakly encephalitogenic in EAE model
Kerlero de Rosbo et al. (31)	1995	Mild pathological signs were detected by inducing MOG_35–55_ in PL/J mice
Mendel et al. (32)	1995	MOG_35–55_ induces highly reproducible EAE in C57BL/6J and C3H.SW (H-2b) mice
Devaux et al. (60)	1997	Severe EAE with truncated human MOG (1–120) in SJL and (PLJ × SJL) F1 mice, encephalitogenic T cell proliferation against epitope MOG_92–106_
Slavin et al. (61)	1998	Relapsing-remitting disease course in NOD/Lt mice (H-2g7) and chronic paralytic disease course in C57BL/6 mice after injection of MOG_35–55_
Weissert et al. (62)	1998	Major histocompatibility complex (MHC) haplotype influences the degree of disease susceptibility, recruitment of MOG-specific immune cells, and pathology in MOG-induced EAE rats
Storch et al. (63)	1998	Immunization with MOG antigen in rats is able to mimic classical multiple sclerosis (MS) as well and variants such as optic neuritis (ON), Devic’s and Marburg’s disease
Encinas et al. (64)	1999	Active immunization with MOG_35–55_ induces relapsing-remitting EAE followed by a secondary progression in NOD mice
Raine et al. (65)	1999	MOG-induced EAE in marmosets lead to vesicular disruption and production of antigen-specific autoantibodies similar to MS
Abdul-Majid et al. (66)	2000	MOG_79–96_ is highly encephalitogenic in DBA/1 mice, including macrophage infiltration and demyelination
Kerlero de Rosbo et al. (67)	2000	rhMOG-EAE induced marmosets with different MHC background showed proliferative T cell responses against epitopes MOG_4–20_, MOG_35–50_, and MOG_94–116_
Bourquin et al. (68)	2000	MOG-DNA vaccination lead to severe EAE
Brok et al. (69)	2000	Human MOG peptide MOG_14–36_ is highly encephalitogenic in marmosets (presented by a common class II Caja-DRB*W1201 molecule)
Weissert et al. (70)	2001	MOG_91–114_ immunization lead to clinical and histopathological EAE signs in LEW.1AV1 and LEW.1N rats
Bettelli et al. (71)	2003	Development of spontaneous ON in T cell receptor (MOG_35–55_) transgenic C57BL/6 mice
Delarasse et al. (14)	2003	MOG-deficient mice are resistant to rat MOG-induced EAE and developed a mild pathological phenotype after immunization of whole myelin. However, B- and T cell responses against the extracellular domain and peptides of MOG were not altered compared to wild-type mice, indicating MOG being resistant to the induction of immune tolerance
Sun et al. (72)	2003	CD8^+^ MOG-specific T cells recognize H-2Db dimers coupled with encephalitogenic peptide MOG_40–54_
Smith et al. (73)	2005	Injection of full-length conformational MOG leads to chronic progressive EAE, but released MOG does not induce immunity during an ongoing disease in Biozzi ABH mice
Krishnamoorthy et al. (74)	2006	MOG_35–55_ leads to paralytic EAE and ON in a double-transgenic (IgH^MOG^ and TCR^MOG^) C57BL/6 line
de Graaf et al. (75)	2008	In LEW.1N, LEW.1AV1, and dark agouti rats, MS-like pathology is mainly determined by presentation of MOG peptides on MHC class II molecules
Kap et al. (76)	2008	Cytotoxic T cells specific to epitope MOG_34–56_ trigger fast progression of rhMOG-induced EAE in marmosets
Matsumoto et al. (77)	2009	MOG_91–108_ is an encephalitogenic epitope able to induce mild T cell-mediated EAE but does not elicit Abs against the epitope or MOG in LEW.1AV1 rats
Pollinger et al. (78)	2009	Development of relapsing-remitting EAE in TCR (MOG_92–106_) transgenic SJL/J mice
Bettini et al. (79)	2009	CD8^+^ T cell dominant epitope MOG_37–46_ lead to mild form of EAE
York et al. (80)	2010	MOG-specific CD8^+^ T cells are able to ameliorate CD4^+^ driven EAE
Anderson et al. (81)	2012	CD4^+^ and CD8^+^ T cell driven EAE in transgenic MOG_35–55_ specific T cell mouse line (1C6)
de Graaf et al. (27)	2012	Correct refolding of MOG increases its encephalogenicity by enhancing its processing or/and presentation on MHC molecules
Jagessar et al. (82)	2012	MOG_34–56_ specific cytotoxic T cells are key regulators for gray and white matter demyelination in marmosets
Delarasse et al. (34)	2013	Transmembrane regions MOG_113–127_ and MOG_120–134_ and second hydrophobic domain MOG_183–197_ are found to be immunogenic and pathogenic in C57BL/6 (H-2b)
Ortega et al. (83)	2013	CD8^+^ cells reactive to MOG_35–55_ attenuate EAE severity in an adaptive CD4 T cell-mediated EAE model in C57BL/6 mice
Haanstra et al. (84)	2013	rhMOG (1–125) induces EAE in non-human primates
Shetty et al. (35)	2014	T cells directed to an encephalitogenic transmembrane domain (MOG_110–132_) induced clinical EAE, inflammation, and demyelination
Curtis et al. (85)	2014	Injection of rat immunoglobuline variable of MOG together with incomplete Freud’s adjuvant lead to atypical EAE in LEW rats and Macaca species
Herrera et al. (86)	2014	MOG_35–55_ induced EAE in C57BL/6 mice lead to lesions along the optic chiasm

Immunizations of LEW rats with MOG activates MOG_1–20_- and MOG_35–55_- specific T cells which are only poorly encephalitogenic (24) and induces MOG-specific Abs which cause formation of focal small demyelinating lesions (24). In contrast to LEW rats are brown Norway and dark agouti rat strains highly susceptible to MOG-induced EAE (87). Different MHC haplotypes and non-MHC background genes modify the anti-MOG immune response (70, 75). This important information derived from EAE studies in MHC congenic LEW rats, i.e., in rats with different MHC class II alleles on the genetic background of LEW rats. Upon immunization with MOG, these animals either develop early onset acute lethal disease with extensive demyelinating plaques, chronic and/or relapsing types of disease, or do not show any evidence of clinical and histological disease, depending on the MHC class II haplotype present (62). Moreover, the MOG-induced T cell proliferation and interferon-gamma production, and the degree of MOG-specific B cell responses and Ab titers correlated with the severity of clinical disease (62). For further experiments, rats were selected which carried the most permissive MHC class II haplotype for the induction of MOG-specific autoimmune reactions, but differed in their non-MHC background genes. When these animals were sensitized with MOG, they mounted anti-MOG T cell and B cell responses, but showed differences in the maturation of these responses (62). Cumulatively, these data suggested that the MHC haplotype influences the degree of disease susceptibility, the clinical course, the recruitment of MOG-specific immunocompetent cells, and the CNS pathology, while non-MHC genes strongly influence the maturation of the anti-MOG response (62). A similar effect was also seen in human HLA DR4 transgenic mice which indicated that HLA DR shaped the anti-MOG response in both, humans and mice (88).

Further knowledge about the role of B cells in MOG-induced CNS inflammation derived from transgenic mice (Table 3). Mice were genetically engineered to express the heavy chain from the monoclonal anti-MOG Ab 8–18C5 described above, paired with endogenous Ig light chains (89). These animals had many MOG-reactive B cells in their immune repertoire and had titers of anti-MOG Abs in their circulation. And yet, they remained completely healthy until they were challenged with MOG. Then, they developed EAE with higher incidence, severity, and earlier onset compared to their non-transgenic counterparts (89). Further studies using B cell-deficient mice showed that B cells are required for EAE induction using the MOG protein, but are dispensable when the encephalitogenic MOG peptide is used for EAE induction (90–92). These studies also revealed that B cells are needed for the recovery from EAE, by the production of IL-10 and expression of CD40 (93). The role of B cells in promoting EAE was further confirmed by using transgenic mouse lines in which MHC class II products were knocked-out in B cells, or in which B cells were able to express MOG-specific B cell receptors on their surface, but were unable to secrete MOG-specific Abs (94). This and several other studies (see Table 3) revealed that B cells can act as APC, and that they can sufficiently promote pro-inflammatory T cell activation and spontaneous EAE onset (91, 94, 95). In another study, in which MOG-specific B cells and T cells were actively transferred into an intact immune repertoire of C57BL/6J mice, MOG-specific B cells were shown to aggravate CNS inflammation and EAE disease course. These results were further confirmed by using human MOG positive serum Abs, reproducing the same disease accelerating effects (58). Hence, both B cells and myelin-specific Abs can independently activate T cells and thus increase the risk of an autoimmune mediated inflammation of the CNS (59).

**Table 3:** **The role of B cells in experimental autoimmune encephalomyelitis (EAE) animal models**.

Reference	Year	Findings
Hjelmstrom et al. (92)	1998	B cell-independent demyelination in myelin oligodendrocyte glycoprotein (MOG)-induced EAE mice
Litzenburger et al. (89)	1998	MOG-specific B cells accelerate and exacerbate EAE, but are not able to induce spontaneous disease or demyelination without induced EAE
Stefferl et al. (87)	1999	Major histocompatibility complex (MHC) and MHC-linked effects can influence the antibody response and thereby disease severity in MOG-induced EAE
Lyons et al. (90)	1999	B-cell-deficient mice immunized with MOG_35–55_ induced EAE but not mice immunized with recombinant full-length MOG
Forsthuber et al. (88)	2001	MOG peptide 97–108 is the immunodominant human leukocyte antigen (HLA)-DR4-restricted T cell epitope in transgenic mice and is presented by human B cells expressing HLA-DR4 (DRB1*0401)
Lyons et al. (91)	2002	MOG-specific B cells and serum reconstitute the ability for inducing inflammatory EAE effects in B cell-deficient mice
Fillatreau et al. (93)	2002	IL-10 production of B cells regulate type 1 immunity and play a key role in EAE recovery
Svensson et al. (96)	2002	B cell-deficient mice with different genetic backgrounds (C57BL/10 and DBA/1) immunized with MOG_1–125_ showed decreased demyelination but inflammation was not affected
Bettelli et al. (97)	2006	TCRMOG × IgHMOG mice develop severe EAE, with inflammatory lesions in the spinal cord and optic nerves
Pollinger et al. (78)	2009	Transgenic mice expressing MOG_92–106_ specific T cells expand endogenous MOG-specific B cells, producing conformational, (epitope independent) Abs, and enhancing demyelinating EAE in a relapsing-remitting EAE model
Molnarfi et al. (94)	2013	MOG-specific B cells play a critical role in the EAE pathogenesis due to its function as an antigen-presenting cells
Parker Harp et al. (95)	2015	B cells directly interact with dendritic cells and enhance CD4 driven EAE severity in mice
Flach et al. (58)	2016	MOG-specific B cells accelerate MOG T cell driven EAE inflammation and disease severity

Also spontaneous models of MOG-induced CNS disease were highly informative for deciphering the role of anti-MOG responses in autoimmune disease. These models were based on the transgenic expression in mice of MOG-specific T cell receptors, either alone (71, 78) or in combination with MOG-specific B cell receptors (74, 97) and gave striking results:

The overexpression of MOG-specific T cell receptors in transgenic C57/BL6 (71) or SJL (78) mice lead to spontaneous optic neuritis (ON) in more than 30% of all animals and rendered the animals hyper-susceptible to the induction of ON in response to sensitization with suboptimal amounts of MOG (71), or to a severe spontaneous relapsing-remitting EAE with episodes often altering between different CNS compartments in more than 60% of all male, and more than 80% of all females within 160 days after birth (78). In these animals, the transgenic T cells expanded MOG-specific B cells from the endogenous immune repertoire, which produced pathogenic autoantibodies binding to a conformational epitope on native MOG protein (78). Overexpression of MOG-specific T cell receptors in NOD mice led to MOG-specific CD4^+^ and CD8^+^ T cell responses at the same time (79). These animals revealed that CD8^+^ MOG-specific T cells may be weakly encephalitogenic (79) and are able to regulate and attenuate CD4^+^ driven immune responses by modulating APC functions and reducing CD4^+^ T cell responses (80, 83).

Mice genetically engineered to express MOG-specific receptors on T and B cells (74, 97) showed a class switch of MOG Abs to an IgG1 subtype, and spontaneously developed inflammatory demyelinating CNS disease (74, 97). Most interestingly, spontaneous development of disease in these animals crucially depended on the presence of commensal microbiota in the gut (98).

Although many seminal observations on MOG-reactive T and B cell responses derive from murine EAE models, it is important to know that in these animals, large lesions with myelin loss are mainly caused by axonal degeneration with secondary demyelination, while primary demyelination is sparse (99, 100). Therefore, it is necessary to also study MOG autoreactivity in the marmoset (*Callithrix jacchus*), in which MOG-induced EAE resembles human demyelinating diseases more closely (100–102). When these animals are immunized with the recombinant IgV domain of rat MOG, they developed lesions which were very similar to chronic multiple sclerosis (MS) plaques with mononuclear cell infiltrates, primary demyelination, and astrogliosis (103), even at the ultrastructural level (65). Moreover, some animals developed a progressive form of EAE, which was triggered by cytotoxic effector memory T cells and further promoted demyelination in the gray matter (76, 82). As seen before in mice and rats, the marmoset CD4^+^ T cell response against MOG may cover several different epitopes, only one of which is highly encephalitogenic (104).

Cumulatively, these animal models revealed that

- autoimmune responses to MOG can be induced in many different species
- the susceptibility to MOG is determined by MHC- and non-MHC genes
- anti-MOG responses typically involve CD4^+^ T cells and complement-fixing Abs of the IgG1 subtype
- the MOG-specific T cell repertoire contains T cells specific for several different T cell epitopes which vary between different species and substrains dependent on the MHC haplotype
- not all MOG-specific T cells are encephalitogenic
- MOG-specific B cells have Ab-dependent and Ab-independent effects on tissue damage
- different types of anti-MOG Abs exist, but only those recognizing conformational epitopes on the extracellular domain of MOG are pathogenic
- MOG-specific autoantibodies in the circulation specific for such conformational epitopes are harmless, unless these Abs gain access to the CNS *via* an opened blood-brain barrier in an inflammatory environment
- MOG-specific Abs can cross-react with other proteins like butyrophilin
- the extent of demyelination caused by anti-MOG Abs depends on MHC-dependent and MHC-independent factors.

## Clinical Relevance of MOG Abs in Demyelinating Diseases

As outlined above, MOG is one of the best-studied autoantigens for experimental autoimmune models for MS. Attempts to translate these findings into the human disease have yielded controversial results, especially with regard to MOG Abs as a prognostic biomarker in MS (105, 106) [reviewed in Berger et al. (107)]. These results were caused by the use of inappropriate methods (e.g., immunoblotting, ELISA) and antigens (recombinant human MOG produced in *Escherichia coli*, MOG peptides) to determine disease-specific MOG Abs. However, with improved detection methods using correctly folded and glycosylated MOG protein expressed in mammalian cells for radioimmunoassays, flow cytometry, and immunofluorescence, MOG Abs were found in a subset of predominantly pediatric patients with acute disseminated encephalomyelitis (ADEM), aquaporin-4 (AQP4) seronegative neuromyelitis optica spectrum disorders (NMOSD), monophasic or recurrent isolated ON, or transverse myelitis (TM), in atypical MS, brainstem encephalitis, and *N*-methyl-d-aspartate receptor-encephalitis with overlapping demyelinating syndromes, but rarely in classical MS (50, 55, 56, 108–176). Since low-titer MOG Abs are often found in MS patients and controls, most of these studies have used either a “high-titer” cut-off or an IgG1 secondary Ab to increase specificity. Like many other autoantibodies, e.g., to AQP4, MOG Abs are therefore only present in rare diseases indicating widely established immunological tolerance to most autoantigens.

**Figure 1:**
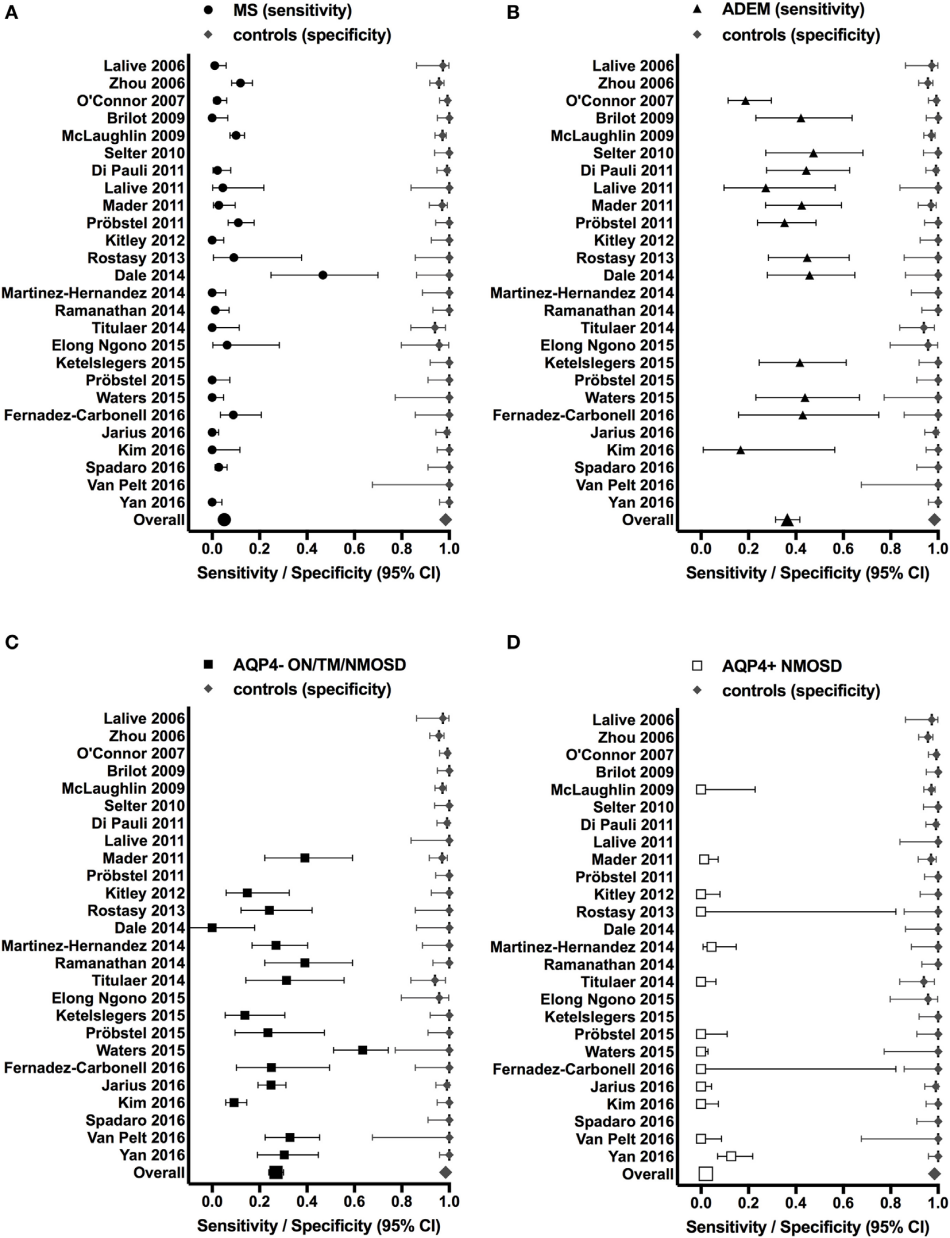
**Studies reporting the presence of myelin oligodendrocyte glycoprotein (MOG) antibodies (Abs) in patients with inflammatory demyelinating disorders (A) MS; (B) ADEM; (C) AQP4− ON/TM/NMOSD; (D) AQP4+ NMOSD (sensitivity, left side of each graph) in comparison to a control group of patients with other neurological disorders and/or healthy controls (specificity, right side of each graph)**. Sensitivities and specificities are indicated by symbols with error bars (95% confidence intervals). Specificities were calculated using the frequency of MOG Abs in other neurological disorders or healthy controls determined by the methods shown in Table 4. Sensitivities were calculated using the frequency of MOG Abs in inflammatory demyelinating disorders determined by the methods shown in Table 4.

These findings, however, raise the important question whether MOG Abs are associated with a specific clinical phenotype like AQP4 Abs are associated with NMOSD (177). We have therefore reviewed the literature and compared all studies, which have analyzed the presence of MOG Abs in inflammatory demyelinating disorders (MS, ADEM, and AQP4 Ab seronegative and seropositive NMOSD) in comparison with a control group of patients with other neurological disorders or healthy controls. Results from these studies are shown in Table 4 and Figure 1. We have identified 26 studies which fulfilled these criteria (50, 55, 56, 109–116, 119, 121, 126, 130, 132, 134, 137, 141, 147, 148, 152, 156, 158, 165, 174). Only 13 of these studies included a control group with 50 or more individuals and only 5 studies included more than 100 controls (Table 4). Further, many patients and controls were repeatedly analyzed in some studies and therefore we decided not to include a statistical analysis of the reviewed publications. The specificity of these studies was calculated using the frequency of MOG Abs in other neurological disorders or healthy controls determined by the methods shown in Table 4. The overall specificity of these studies was 98.5% [95% confidence interval (CI) 97.8–99] and thus 1.5% (range 0–6%) of all controls were seropositive for MOG Abs (Table 4; Figure 1). The sensitivity of these studies was calculated using the frequency of MOG Abs in inflammatory demyelinating disorders determined by the methods shown in Table 4. The presence of MOG Abs in MS was analyzed in 23/26 studies and the overall sensitivity for MS was 5.1% (95% CI 4.2–6.1) and thus 5.1% (range 0–46.7%) of all MS patients were seropositive for MOG Abs. The highest frequency of MOG Abs within MS patients was found in pediatric MS patients and in one of the initial studies not using a high-titer cut-off. Therefore, it can be concluded that MOG Abs are rare in MS, particularly in adult MS, but are still found in a few patients in several studies. Since MOG Abs are associated with MS-like neuropathology (136, 149, 167, 172, 178, 179), they might play a role in pathophysiology in these patients and therefore the current practice to use MS as a negative control group for MOG Abs (141) should be regarded with caution. The presence of MOG Abs in ADEM was analyzed in 13/26 studies and the overall sensitivity for ADEM was 36.4% (95% CI 31.4–41.7; range 17.7–47.4%) and thus ADEM was the most frequent clinical presentation associated with MOG Abs. Again, the frequency of MOG Abs was highest in pediatric patients. Since the 26 studies used different clinical criteria for NMOSD, we reviewed the studies for the presence of MOG Abs in AQP4 seronegative patients with ON, TM, or NMOSD. The presence of MOG Abs in these conditions was analyzed in 15/26 studies and the overall sensitivity was 26.9% (95% CI 23.9–30.1; range 9.2–63.5%). Finally, the presence of MOG Abs in AQP4 seropositive NMOSD was analyzed in 13/26 studies and the overall sensitivity was 2% (95% CI 1.2–3.4; range 1.2–3.4%). Thus, the presence of MOG Abs in AQP4 Ab-positive NMOSD is in the range of the control group.

**Table 4:** **Studies reporting the presence of myelin oligodendrocyte glycoprotein (MOG) antibodies (Abs) in patients with inflammatory demyelinating disorders in comparison to a control group of patients with other neurological disorders and/or healthy controls**.

Reference	Method	Patients	Multiple sclerosis	Acute disseminated encephalomyelitis	Aquaporin-4 (AQP4)− optic neuritis/transverse myelitis/neuromyelitis optica spectrum disorders (NMOSD)	AQP4+ NMOSD	Controls
Lalive et al. (109)	FACS	ad	1/92 (1%)	n.a.	n.a.	n.a.	1/37 (3%)
Zhou et al. (50)	FACS	ad	25/210 (12%)	n.a.	n.a.	n.a.	8/187 (4%)
O’Connor et al. (110)	RIA	ad, ped	3/140 (2%)	13/69 (19%)	n.a.	n.a.	1/133 (1%)
Brilot et al. (112)	FACS	ad, ped	0/54 (0%)	8/19 (42%)	n.a.	n.a	0/73 (0%)
McLaughlin et al. (111)	FACS	ad, ped	39/385 (10%)	n.a.	n.a.	0/13 (0%)	6/214 (3%)
Selter et al. (113)	FACS	ped	n.a.	9/19 (47%)	n.a.	n.a.	0/58 (0%)
Di Pauli et al. (115)	IF-HT	ad, ped	2/89 (2%)	12/27 (44%)	n.a.	n.a.	1/105 (1%)
Lalive et al. 2011 (114)	FACS	ped	1/22 (5%)	3/11 (27%)	n.a.	n.a.	0/20 (0%)
Mader et al. (55)	IF-HT	ad, ped	2/71 (3%)	14/33 (42%)	9/23 (39%)	1/75 (1%)	3/101 (3%)
Probstel et al. (116)	FACS	ad, ped	14/127 (11%)	19/54 (35%)	n.a.	n.a.	0/63 (0%)
Kitley et al. (119)	IF	ad	0/75 (0%)	n.a.	4/27 (15%)	0/44 (0%)	0/23 (0%)
Rostasy et al. (121)	IF-HT	ped	1/11 (9%)	13/29 (45%)	7/29 (24%)	0/2 (0%)	0/23 (0%)
Dale et al. (56)	FACS	ped	7/15 (47%)	11/24 (46%)	13/24 (54%)	n.a.	0/24 (0%)
Martinez-Hernandez et al. (134)	IF-HT	ad	0/64 (0%)	n.a.	14/52 (27%)	2/45 (4%)	0/30 (0%)
Ramanathan et al. (130)	FACS	ad	1/76 (1%)	n.a.	9/23 (39%)	n.a.	0/52 (0%)
Elong Ngono et al. (132)	IF-HT	ad	1/16 (6%)	n.a.	n.a.	n.a.	1/24 (4%)
Ketelslegers et al. (147)	FACS	ped	n.a.	10/24 (42%)	4/29 (14%)	n.a.	0/44 (0%)
Probstel et al. (137)	FACS	ad	0/48 (0%)	n.a.	4/17 (24%)	0/31 (0%)	0/39 (0%)
Waters et al. (141)	IF-IgG1	ad	0/76 (0%)	7/16 (44%)	40/63 (64%)	0/130 (0%)	0/13 (0%)
Fernandez-Carbonell et al. (152)	FACS	ped	4/45 (9%)	3/7 (43%)	4/14 (29%)	0/2 (0%)	0/23 (0%)
Jarius et al. (174)	IF-HT	ad, ped	0/139 (0%)	n.a.	50/202 (25%)	0/83 (0%)	1/98 (1%)
Kim et al. (148)	IF-IgG1	ad	0/29 (0%)	1/6 (17%)	15/163 (9%)	0/49 (0%)	0/72 (0%)
Spadaro et al. (165)	FACS	ad	5/181 (3%)	n.a.	n.a.	n.a.	0/39 (0%)
van Pelt et al. (158)	FACS	ad	n.a.	n.a.	20/61 (33%)	0/41 (0%)	0/8 (0%)
Overall			106/196 (5%)	123/338 (36%)	193/727 (27%)	3/515 (1%)	22/1527 (1%)

*The percentage of MOG Ab seropositivity was determined using the methods indicated in the table*.

*ad, adult; ped, pediatric; n.a., not analyzed; FACS, fluorescence-activated cell sorting; IF, immunofluorescence assay; IF-HT, immunofluorescence assay with high-titer cut-off; IF-IgG1, immunofluorescence assay for IgG1 Abs; RIA, radio immunoprecipitation assay*.

In conclusion, these studies revealed that MOG Abs are associated with heterogeneous clinical presentations and a younger age of onset in human inflammatory demyelinating diseases but a clear common clinical phenotype is missing.

The histopathology associated with MOG Abs has been described in few patients including NMOSD, atypical demyelination, CIS, and ADEM (136, 149, 167, 172, 178, 179) (Table 5). All cases showed demyelinating lesions with features of MS pattern II, with well-demarcated confluent plaques with loss of myelin, relative preservation of axons, well-preserved astrocytes, and numerous macrophages containing myelin debris. The inflammatory infiltrates were predominantly composed of perivascular and parenchymal T-cells and some perivascular B-cells. Moreover, the deposition of terminal complement complex C9neo was reported indicating complement-dependent cytotoxicity (136, 167). All lesions were characterized by well-preserved oligodendrocytes that were partly MOG-negative, most likely compatible with preoligodendrocytes. Demyelination associated with MOG Abs differs from AQP4 seropositive NMOSD that characteristically shows loss of astrocytes with deposition of IgG and terminal complement complex C9neo, inflammatory infiltrates including the presence of neutrophilic and eosinophilic granulocytes, and elevated glial fibrillary acidic protein levels in CSF (180).

**Table 5:** **Neuropathological findings in patients with myelin oligodendrocyte glycoprotein (MOG) antibody (Ab)-associated demyelination**.

Reference	Number of cases	Sex, age (years)	Clinical presentation	Findings
Konig et al. (178)	1	F, 49	RRMS	Multiple sclerosis (MS) pattern II; oligodendrocytes in lesion preserved (CNPase^+^; MOG not determined)
Spadaro et al. (136)	1	F, 66	Recurrent myelitis + brainstem involvement	MS pattern II; oligodendrocytes preserved (CNPase^+^; MOG^−^)
Di Pauli et al. (149)	1	M, 71	Acute disseminated encephalomyelitis (ADEM)/acute MS	MOG and aquaporin-4 Ab positive; MS pattern II; oligodendrocytes preserved (CNPase^+^, MOG^−^)
Jarius et al. (172)	1	F, 63	CIS	MS pattern II; oligodendrocytes preserved (CNPase^+^, MOG^+^)
Wang et al. (167)	1	F, 67	Neuromyelitis optica spectrum disorders	Pattern classification not done; well-demarcated demyelinating lesion with preserved axons and astrocytes
Körtvélyessy et al. (179)	2	M, 49	ADEM	Intrathecal MOG Ab synthesis; MS pattern II; one patient with overlapping features of pattern III (early MAG loss, apoptotic oligodendrocytes in addition to complement deposition)
		M, 34		

These similar immunopathological findings compatible with MS pattern II supports a humoral immune pathogenesis in patients with MOG Abs. Since the histopathological lesion type is independent from the clinical presentation the demyelinating lesions may be included under the term “MOG antibody syndrome.”

## Epitope Recognition and Species Specificity of Human MOG Abs

As MOG Ab binding has been shown to be dependent on the correct folding and glycosylation pattern of their antigen, studies were directed toward the binding motifs/epitopes of these Abs with the aim to identify specific binding patterns for diseases. Mayer and colleagues (122) performed epitope recognition studies of MOG Abs from several demyelinating diseases and seven distinct binding patterns were found. However, no clinical correlation between the binding patterns and different disease entities could be shown. Furthermore, these Abs were directed against only a single epitope or multiple epitopes and an association between glycosylation and an increased binding capacity could not be detected. The most frequent epitopes were found in the CC′-loop and FG-loop of the extracellular IgV domain of correctly folded human MOG protein. Within the CC′-loop, AA P42 was essential for binding and therefore human MOG Abs did not bind either to rodent MOG, which has a serine at position 42, or to mutated human MOG P42S (122). These findings were confirmed and extended by Sepulveda et al. (166) who demonstrated that only a subset of human MOG Abs is also reactive to rodent MOG epitopes as analyzed by cell-based assays and tissue immunohistochemistry and this reactivity to rodent MOG did not correlate with a specific clinical phenotype. Finally, it has been already demonstrated that species differences of MOG lead to the activation of different pathogenic mechanisms in EAE induced with rodent or human MOG_35–55_ or recombinant MOG (26, 92, 181).

## MOG Abs: Epiphenomenon or Indicative for Disease Phenotype

The animal experiments described above clearly indicated that murine MOG Abs can be pathogenic. Furthermore, pathologic similarities to ADEM have been shown in transgenic MOG-IgG mice infected with several neurotrophic encephalitogenic viruses, exacerbating virus-induced CNS inflammation. These similarities were indicated by clinical defined extensive perivascular infiltrates (mixed inflammatory cell population, e.g., lymphocytes, neutrophils, NK cells, and blood born macrophages) and perivenous demyelination (182, 183).

By contrast, only four studies aimed to investigate the pathogenic role of human MOG Abs *in vivo*. Whereas several studies indicated that human MOG Abs can activate complement and cellular-dependent cytotoxicity (50, 55, 112) *in vitro*, these mechanisms were not observed after transfer of human MOG Abs to rodents *in vivo*: the injection of concentrated serum samples from MOG Ab-positive patients into LEW rats with EAE did not increase the clinical score of the disease, but led to a minor increase in demyelination and axonal loss (50). Intrathecal injection of purified human MOG IgG caused reversible brain edema and myelin loss with very little complement deposition at the lesion site (57).

A different pathogenic mechanism for MOG Abs was proposed in two recent studies (58, 59). In the first study (58), it was demonstrated that MOG-specific B cells and their products (MOG Abs) activate MOG-specific effector T cells *via* CNS resident APC. A similar effect was demonstrated for peripheral APC in the second study (59). Both studies emphasize an important role for Ab-mediated antigen opsonization and accumulation in Fc receptor expressing APCs and subsequent increased antigen presentation and activation of specific T cells.

## Are MOG Abs a Primary or a Secondary Immune Response?

The findings discussed in the previous chapter raise the important question whether human MOG Abs are pathogenic themselves or just a epiphenomenal bystander or a secondary immune reaction due to previous demyelination (184). An example for a secondary immune response was shown in a study using a transgenic myelin-specific T cell mice model, which developed spontaneous EAE (98). In this model, an interaction between MOG-specific T and B cells is necessary for inflammatory demyelination, resulting in the activation of native B cells by dendritic cells presenting MOG peptides in the cervical lymph nodes (78). In a gut germ free environment, autoreactive T cell activation failed, and therefore the signal cascade for producing autoantibodies producing B cells was significantly reduced, but increased after microbial re-colonization. One potential mechanism mediating the onset of spontaneous EAE is molecular mimicry, activating encephalitogenic T cells, with subsequent inflammation of the CNS and second, it leads to an activation of native MOG-specific B cells recruited to the CNS tissue *via* locally produced MOG material or drained into the CNS along peripheral lymph nodes.

But even if MOG Abs would only be a secondary immune reaction they still could be clinically relevant biomarkers such as seen in diabetes type I, an autoimmune disease affecting insulin producing β-cells in the pancreas. Four autoantibodies to insulin (185), glutamic acid decarboxylase (186), Islet antigen-2 (187), and zinc transporter 8 (188) have been identified as highly specific biomarkers to predict this disease. There is more than an 80% probability of developing diabetes in children and adolescents, if 2/4 autoantibodies are detected [reviewed in Bonifacio (189)]. However, these autoantibodies are not pathogenic itself, but rather indicate a disturbed immune activity or an underlying T cell-mediated autoimmune process (190). Similarly, it could be that human MOG Abs play only a minor role in the pathophysiology of inflammatory demyelination, but are highly specific markers for affected patients.

## Conclusion

In the past years, autoantibodies emerged as important biomarkers in neurological autoimmune diseases. One of the best examples for these biomarkers is AQP4 Abs as diagnostic marker for NMOSD. Numerous studies have now established a possible similar role for MOG Abs that are associated with a very heterogeneous age-dependent clinical presentation and MS-like neuropathology. The exact pathologic effect of human MOG Abs is still unclear and needs to be critically investigated in order to clarify the immunopathological role of these Abs.

## Author Contributions

PP prepared the main body of the manuscript and tables. MB and TB participated in the preparation of the manuscript. RH participated in the preparation of the manuscript and prepared tables and figures. MR supervised the work and participated in the preparation of the manuscript and figures and tables. All authors approved the final version of the manuscript.

## Conflict of Interest Statement

The Neurological Research Laboratory (Medical University of Innsbruck and Tirol Kliniken) receives payments for antibody assays (AQP4- and anti-neuronal antibodies) and for MOG and AQP4 antibody validation experiments organized by Euroimmun (Germany).

## Abbreviations

AA, amino acids; Ab, antibody; ADEM, acute disseminated encephalomyelitis; AQP4, aquaporin-4; BN, brown Norway; CNS, central nervous system; CSF, cerebrospinal fluid; DA, dark agouti; EAE, experimental autoimmune encephalomyelitis; HLA, human leukocyte antigen; Ig, immunoglobulin; MBP, myelin basic protein; MHC, major histocompatibility complex; MOG, myelin oligodendrocyte glycoprotein; MS, multiple sclerosis; NMOSD, neuromyelitis optica spectrum disorders; ON, optic neuritis; TM, transverse myelitis.
